# Sucrose Facilitates Rhizome Development of Perennial Rice (*Oryza longistaminata*)

**DOI:** 10.3390/ijms232113396

**Published:** 2022-11-02

**Authors:** Zhiquan Fan, Guanwen Huang, Yourong Fan, Jiangyi Yang

**Affiliations:** State Key Laboratory for Conservation and Utilization of Subtropical Agro-Bioresources, College of Life Science and Technology, Guangxi University, Nanning 530004, China

**Keywords:** *Oryza longistaminata*, rhizome, sucrose, RNA-Seq, transcriptome

## Abstract

Compared with annual crops, perennial crops with longer growing seasons and deeper root systems can fix more sunlight energy, and have advantages in reducing soil erosion and saving water, fertilizer and pesticide inputs. Rice is one of the most important food crops in the world. Perennial rice can be of great significance for protecting the ecological environment and coping with the shortage of young farmers due to urbanization. *Oryza longistaminata* (*OL*) is a rhizomatous wild rice with an AA genome and has strong biotic and abiotic resistances. The AA genome makes *OL* easy to cross with cultivated rice, thus making it an ideal donor material for perennial rice breeding. Sucrose plays an important role in the development and growth of plants. In this study, *OL* seedlings were cultured in medium with different concentrations of sucrose, and it was found that sucrose of appropriate concentrations can promote the sprout of basal axillary buds and the subsequent development of rhizomes. In order to explore the molecular mechanism, comparative transcriptome analysis was carried out with *OL* cultured under two concentrations of sucrose, 20 g/L and 100 g/L, respectively. The results showed that the boost of sucrose to rhizome elongation may be due to the glucose and fructose, hydrolyzed from the absorbed sucrose by vacuolar acid invertase. In addition, the consequent increased osmotic pressure of the cells would promote water absorption, which is benefit for the cell elongation, eventually causing the rhizome elongation. These results may provide a reference for elucidating the regulatory mechanism of sucrose on the rhizome development of *OL*.

## 1. Introduction

*O. longistaminata* (*OL*) is a perennial wild rice with well-developed rhizomes. Rhizome is the key organ for vegetative reproduction and perenniality of *OL* [1,2,3,4,5]. The rhizome of *OL* is a horizontally growing underground stem that can grow out of the soil and become a new plant (ramet). Axillary buds on the nodes of the rhizome can produce secondary rhizomes or new ramets (new plants genetically identical to the parent). In addition, the rhizome is also an important storage organ of water and nutrients, such as starch [6], which is of great significance for *OL* to survive through harsh environments.

The development of rhizome in *OL* is a complex trait, maybe controlled by single, dual or multiple genes. A single dominant allele *Rhz* on chromosome 4 was firstly proposed for the rhizomatous growth habit in *OL*, and modifying genes have also been suggested to be involved for the various *Rhz* phenotypes among F_2_ plants [7]. A pair of dominant complementary genes, *Rhz2* and *Rhz3,* have subsequently been identified for the regulation of rhizomes in *OL* [4]. However, only *Rhz2* and *Rhz3* did not guarantee the presence of rhizomes in the hybrid progeny between cultivated rice and *OL* (Hu, 2015, presentation entitled “Progress in Perennial Rice Breeding and Genetics”, accessible from http://pwheat.anr.msu.edu/2015/02/ (accessed on 25 September 2022)). Thirteen QTLs (quantitative trait loci) regulating rhizome development have recently been mapped by Fan, et al. [8]. Further study from Hu’s group identified 13 major-effect loci that jointly control rhizomatousness in *OL*, and as many as 51 QTLs for the rhizome abundance [9]. On the whole, the development of rhizome is regulated by multiple genes [8,9,10].

In addition to the genetic factor, environmental factors, such as sugars, also play an important role in rhizome development [6,11]. Sucrose can promote secondary rhizome development from the axillary buds of isolated rhizomes [6,11]. The rhizome usually grows horizontally below the ground for a certain distance, and then grows upward due to the negative gravitropism, and finally grows out of the soil and becomes a new ramet. It has been found that sucrose can retard the upward growth of rhizomes [6,11], causing speculation that sucrose is the key stimulus inducing the development and transformation of rhizomes [6]. However, the molecular mechanism by which sucrose affects rhizome development in *OL* remains unclear. In the previous studies, the isolated rhizomes were all cultured with sucrose in vitro [6,11], and the valid experiment about the effect of sucrose in rhizome development should be carried out in vivo. In this study, *OL* seedling was cultured in solid medium with different concentrations of sucrose to observe the effects of sucrose on the formation and development of rhizomes. In addition, the regulatory network of rhizome development in *OL* was probed by comparative transcriptome analysis.

## 2. Results

### 2.1. Sucrose Is Important for Sprouting of the Axillary Buds and Rhizome Growth in O. longistaminata (OL)

**Figure 1:**
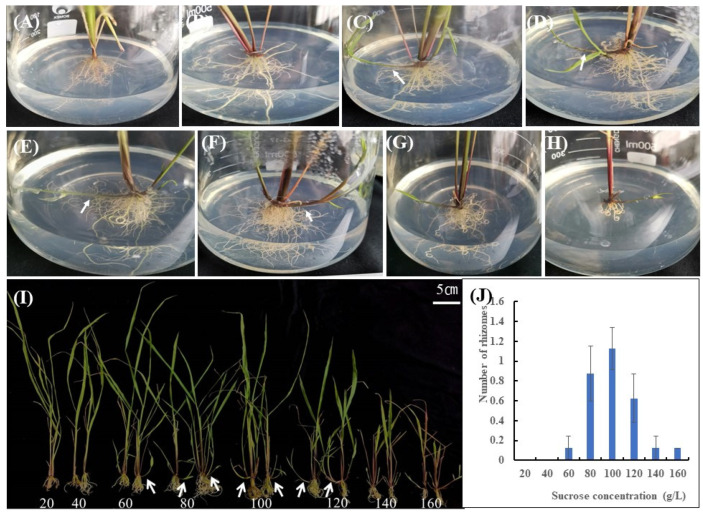
Effects of different sucrose concentrations on rhizomes of *Oryza longistaminata*. The corresponding sucrose concentrations in (**A**–**H**) are 20 g/L (**A**), 40 g/L (**B**), 60 g/L (**C**), 80 g/L (**D**), 100 g/L (**E**), 120 g/L (**F**), 140 g/L (**G**) and 160 g/L (**H**), respectively. Rhizomes are indicated with white arrows. (**I**) Phenotypes of the whole plants cultivated with various concentrations of sucrose. In (**J**), the abscissa represents different sucrose concentrations, and the ordinate represents the average number of rhizomes per plant under the corresponding sucrose concentration. The error bars represent standard deviation.

In order to explore the effect of sucrose on rhizome development, the aseptic *OL* seedlings at three–four leaf stage were cultured with different concentration of sucrose. It was found that when the sucrose concentration was 20 g/L, there are no axillary bud sprouted (Figure 1A). With the increase of sucrose concentration, such as to 40 g/L, a few sprouted axillary buds were found to form tillers or rhizomes (Figure 1B). When sucrose concentration rose to 60 g/L, the axillary buds of all seedlings sprouted, with many rhizomes formed (Figure 1C). At 80 g/L and 100 g/L, a large number of axillary buds sprouted to form rhizomes (Figure 1D,E). Compared with other concentrations, longer and more robust rhizomes were found at these two sucrose concentrations, with a more pronounced effect observed at the 100 g/L group. With further increase of sucrose concentration, the positive effect of sucrose on rhizome development became less prominent. At 120 g/L sucrose, even though the plant growth was slightly inhibited, rhizomes still could be found (Figure 1F). When the sucrose concentration increased to 140 g/L or 160 g/L, a harmful effect appeared and the sprouted axillary buds would turn up quickly (Figure 1G,H), with the leaves withered, maybe due to the high-water stress (Figure 1I). The results showed that a certain concentration of sucrose promotes the sprouting of axillary buds and the formation of rhizomes in *OL*.

### 2.2. Seedling Culture for Transcriptome Sequencing

**Figure 2:**
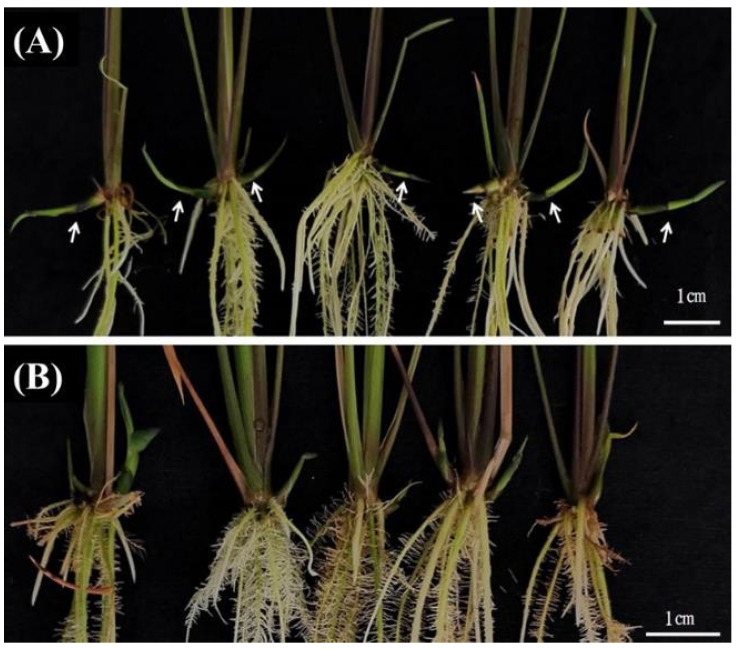
Branch formation in mediums with different sucrose concentrations and additional uniconazole. The working concentration of uniconazole is 0.2 mg/L. The sucrose concentration in (**A**) is 100 g/L, and that in (**B**) is 20 g/L. Arrows indicate rhizomes.

In order to explore the molecular mechanism of sucrose affecting the development of *OL* rhizomes, crowns (the shortened internodes at stem base, also called shortened basal internodes), together with attached axillary buds, were collected for transcriptome sequencing. Usually, when cultivated rice seedlings are grown in a medium containing 20 g/L sucrose, axillary buds can sprout. However, when 20 g/L sucrose was used to culture *OL*, there was no axillary bud sprouted (Figure 1A). Therefore, a certain concentration of uniconazole, which can promote the sprouting of axillary buds [12], was added to the sucrose medium to promote sprouting of axillary buds. According to the effects of different uniconazole concentrations on the plant height, axillary bud sprouting and rhizome development, the optimum concentration of uniconazole was determined. The optimum concentration of uniconazole was 0.2 mg/L. If the concentration of uniconazole was too low, the sprouting of axillary buds in 20 g/L sucrose group was rare, which is not appropriate for the observation of the development of axillary buds. When uniconazole concentration was too high, although it could fully induce the sprouting of axillary buds, the plants were too short and this was able to surpass the promoting effect of 100 g/L sucrose on rhizome growth, resulting in the development of all axillary buds into tillers in both the 20 g/L and 100 g/L sucrose groups. When the concentration of uniconazole was 0.2 mg/L, the sprouted axillary buds in the 100 g/L sucrose group could develop into both tillers and rhizomes (Figure 2A); however, compared with the same concentration of sucrose without uniconazole (Figure 1E), the rhizomes of plants with uniconazole (Figure 2A) were shorter. However, at the concentration of 0.2 mg/L uniconazole, although the axillary buds of the 20 g/L sucrose group could sprout, they almost all developed into tillers (Figure 2B). Therefore, the crowns, together with attached axillary buds in the 20 g/L sucrose plus 0.2 mg/L uniconazole, were taken as the control group, denoted S20, and the same parts in the 100 g/L sucrose plus 0.2 mg/L uniconazole were the treatment group, denoted S100. RNA samples of both S20 and S100 were committed to transcriptome sequencing.

### 2.3. Sample Correlation Analysis

Based on the Illumina Nova seq 6000 sequencing platform, all samples were sequenced according to the instructions. The original sequencing data (raw data) were trimmed to remove low-quality and short sequence data, resulting in a total of 47.94 Gb of clean data in six samples; the clean data of each sample totaled over 7.66 Gb, and the percentage of Q30 bases was over 94.81% (Table S3). The clean reads of each sample were aligned with the reference genome, and the mapped rates ranged from 81.39% to 82.56% (Table S3).

In order to test whether the variation between biological repeats was in line with the expectations of the experimental design, and to provide a basic reference for differential gene analysis, the TPM (transcripts per million reads) value was used as the gene expression level, the correlation analysis of each biological repeat was carried out based on the Pearson correlation coefficient and the method of complete linkage was used for clustering. The results showed that the correlation coefficients between biological replicates in this study were all greater than 0.97 (Table S4), and the samples of biological replicates were clustered together (Figure S1). The above results show that the sampling was reasonable, and all sample data can be used for the subsequent analysis. In the study, a total of 38,866 genes were detected. These genes were aligned with six major databases (NR, Swiss-Prot, Pfam, EggNOG, GO and KEGG) to obtain gene functional information (Table S5). Given that the genome of *O. sativa* was used as the reference genome, all interpretations of gene functions are based on the annotation of *O. sativa* gene.

### 2.4. Functional Analysis of Differentially Expressed Genes

TPM (transcripts per million reads) was used as a quantitative index to analyze the quantitative results of gene expression. Based on the relative expression of genes, with |log_2_FC| ≥ 1 and *P-adjust* < 0.05 as the threshold, 1255 differentially expressed genes (DEGs) with significance were obtained between S20 and S100 groups (Table S5). Compared with the S20 group, 529 were up-regulated and 726 were down-regulated in the S100 group (Figure S2). DEGs were classified and analyzed by COG (cluster of orthologous groups of proteins), GO (gene ontology) and KEGG (Kyoto Encyclopedia of Genes and Genomes).

**Figure 3:**
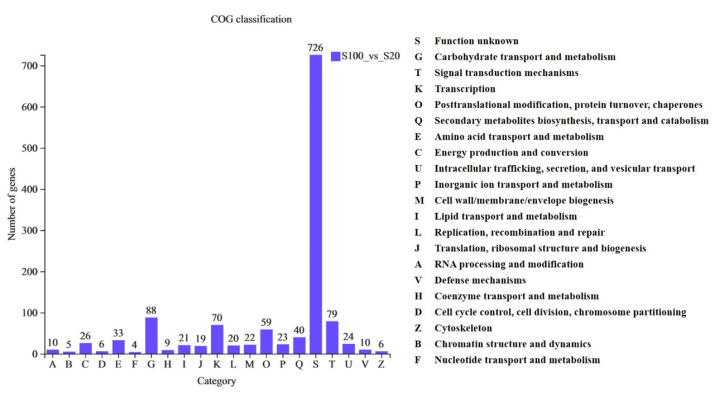
Histogram of COG classification statistics of differentially expressed genes. The abscissa represents the functional type of COG, and the ordinate represents the number of genes with this type of function.

It was found that, except for genes with unknown COG functions, the most functional one was G type (carbohydrate transport and metabolism) (Figure 3), and this functional type contains 88 genes (Table S6), of which *Os10g0555651* and *Os10g0555700* (*OsEXPB2*) were described as being able to cause loosening and extension of plant cell walls by disrupting non-covalent bonding between cellulose microfibrils and matrix glucans. *Os10g0577500* and *Os10g0545500* were annotated as xyloglucan endotransglucosylase hydrolase protein gene, and *Os06g0725300* as an expansin-like gene. *Os01g0249100* and *Os01g0249050* were plant-type cell wall organization genes. *Os09g0530250* was endoglucanase. *Os01g0813800*, *Os05g0365700* and *Os01g0940800* were beta-glucosidase. *Os03g0300600* was pectinesterase. In addition, nine genes, including *Os02g0106100* (*OsINV3*), were described as hydrolases. This indicated that some genes involved in cell wall loosening and extension, hydrolysis and remodeling of cell wall components were induced by sucrose. It means that sucrose treatment induced the loosening and extension of plant cell walls, and the hydrolysis and remodeling of cell wall components. In addition, these 88 genes included some genes related to aquaporin proteins, such as *Os07g0448100* (*OsPIP2;4*), *Os01g0975900* (*OsTIP1;2*), *Os07g0448150*, *Os05g0231700*, *Os02g0658100* (*OsTIP2;1*), *Os04g0521100*, *Os06g0552900*, *Os06g0553001*, *Os04g0233851*, *Os07g0448200* and *Os07g0448600,* which were described as channels that permit osmotically driven movement of water in both directions and are involved in the osmoregulation and in the maintenance of cell turgor during volume expansion in rapidly growing cells. It indicates that sucrose treatment induces the change of osmotic pressure of plant cells.

**Figure 4:**
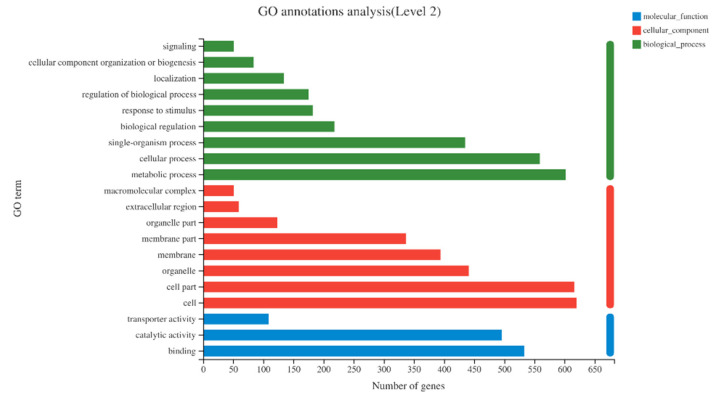
Histogram of GO classification statistics of differentially expressed genes. The vertical axis in the figure represents the secondary classification term of GO, and the horizontal axis represents the number of genes in the secondary classification.

GO annotation was performed on these 1255 DEGs and these genes were classified into 49 GO items. These included 21 biological_process entries, 15 cellular_component entries and 13 molecular_function entries (Figure 4). Biological_process had the largest number of DEGs in the metabolic process (GO:0008152), followed by cellular process (GO:0009987). Cellular_component had the largest number of DEGs in the cell (GO:0005623), followed by cell part (GO:0044464). Molecular_function had the largest number of DEGs in binding (GO:0005488), followed by catalytic activity (GO:0003824) (Figure 4).

**Figure 5:**
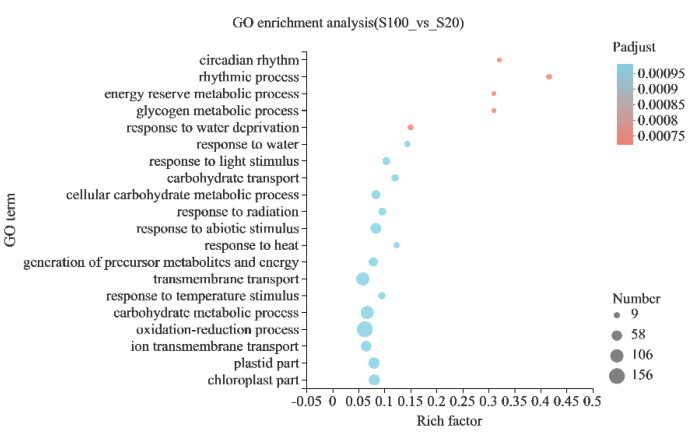
GO enrichment analysis. The vertical axis is the GO entry and the horizontal axis is the ratio of the enriched gene number (sample number) to the annotated gene number (background number) in the entry. The larger the ratio, the higher the enrichment degree. The size of the dot represents the number of genes in the entry; the larger the dot, the more genes there are. The color of the dot shows different *P−adjust* values; the smaller the *P−adjust*, the redder the dot. The figure shows the enrichment results of the top 20 entries of *P−adjust* from small to large.

The DEGs were further analyzed for GO functional enrichment to obtain the main GO functions of the genes. When *P-adjust* < 0.05, this GO item was considered to be significantly enriched. It was found that a total of 125 GO items were significantly enriched, including circadian rhythm (GO:0007623), energy storage and metabolism (GO:0006112), carbohydrate transport and metabolism (GO:0008643, GO: 0044262, GO: 0005975), responses to water (GO: 0009414, GO: 0009415), etc. (Figure 5). Among them, there were 130 genes involved in carbohydrate transport and metabolism (Table S7). Many of these genes were the same as the genes in the G functional classification of COG, including vacuolar acid invertase gene *Os02g0106100* (*OsINV3*) and xyloglucan endotransglucosylase hydrolase protein genes *Os10g0545500*, *Os06g0697000* and *Os10g0577500*. There were also some other cell wall component-related genes, such as pectinesterase (*Os11g0172100*), polygalacturonase (*Os01g0636500*, *Os05g0279900*, *Os05g0279850*, *Os05g0578750*, *Os05g0542900*, *Os01g0296200*, *Os05g0578600* (*PSL1*), *Os01g0329300*) and endoglucanase (*Os09g0530250*, *Os08g0387400*). In addition, there were some glycosyltransferase genes, sugar transporter genes, amylase genes, sucrose synthase genes and starch synthase genes among these 130 genes, such as *Os03g0170900* (*OsSUT1*), *Os07g0616800* (*RSUS3*; *SUS3*), *Os07g0106200* (*OsMST3*), *Os02g0513100* (*OsSWEET15*) and *Os06g0160700* (*OsSSI*). There were 17 genes that responded to water (Table S8), mainly related to drought stress, such as catalase gene *Os02g0115700* (*OsCATA*) [13], aquaporin protein gene *Os07g0448100* (*OsPIP2;4*), *Os04g0521100*, *Os02g0823100* (*OsPIP1;3*), *Os07g0448200*, ABA-related genes *Os05g0213500* (*OsPYL/RCAR5*; *OsPYL5*), *Os11g0167800* (*OsASR1*), *Os11g0454300* (*RAB21*; *Rab16A*) and transcription factor *Os03g0741100* (*OsbHLH148*), which regulates drought tolerance in rice [14].

**Figure 6:**
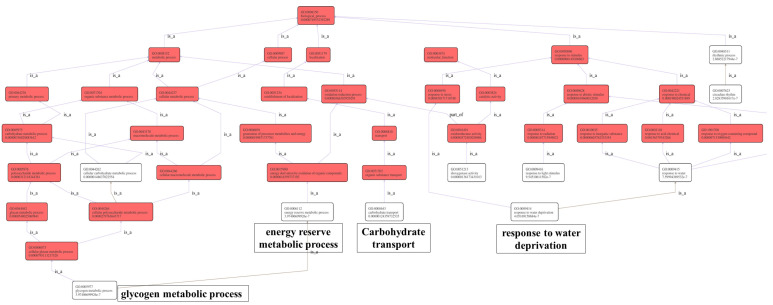
GO directed acyclic graph. The boxes show the GO IDs and annotations of the GO items. Red indicates the GO items with significant enrichment; the redder the color, the more significant the enrichment. The connecting line indicates the relationship between the two GOs. Please refer to http://geneontology.org/docs/ontology-relations/ (accessed on 25 September 2022) for the relationship explanation.

Genes in the GO enrichment analysis results were used for the GO directed acyclic graph analysis, which shows the relationship between the upper and lower levels of the GO items and the degree of enrichment. It was found that these GO items were mainly divided into two branches, namely carbohydrates metabolism and response to water deprivation (Figure 6). In addition, there were three minor branches, namely carbohydrate transport, dioxygenase activity and response to light stimulation (Figure 6). Carbohydrate transport and metabolism were involved in the utilization of high concentrations of sucrose and the response to water deprivation, suggesting that high concentrations of sucrose may lead to increased intracellular osmotic pressure.

**Figure 7:**
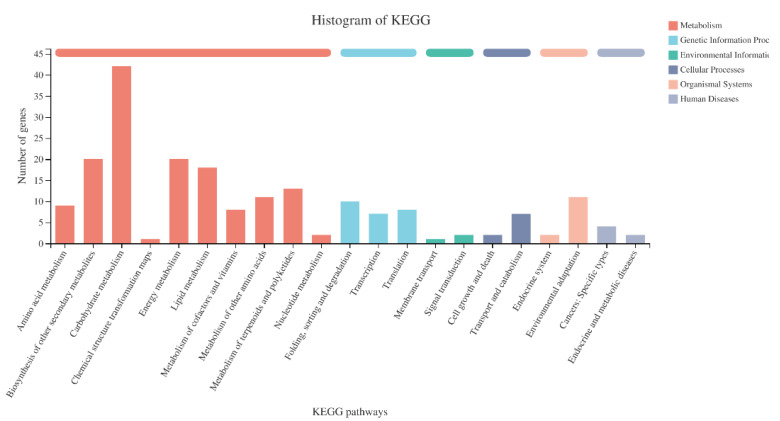
Classification statistics of differentially expressed genes in the KEGG pathway. The abscissa is the name of the KEGG metabolic pathway; the ordinate is the number of genes annotated to this pathway.

KEGG annotation analysis of DEGs found that there were 5 first-level KEGG pathway categories and 21 s-level KEGG pathway categories. Among them, the DEGs in the classification of carbohydrate metabolism were the most numerous ones, followed by biosynthesis of other secondary metabolites and energy metabolism (Figure 7).

**Figure 8:**
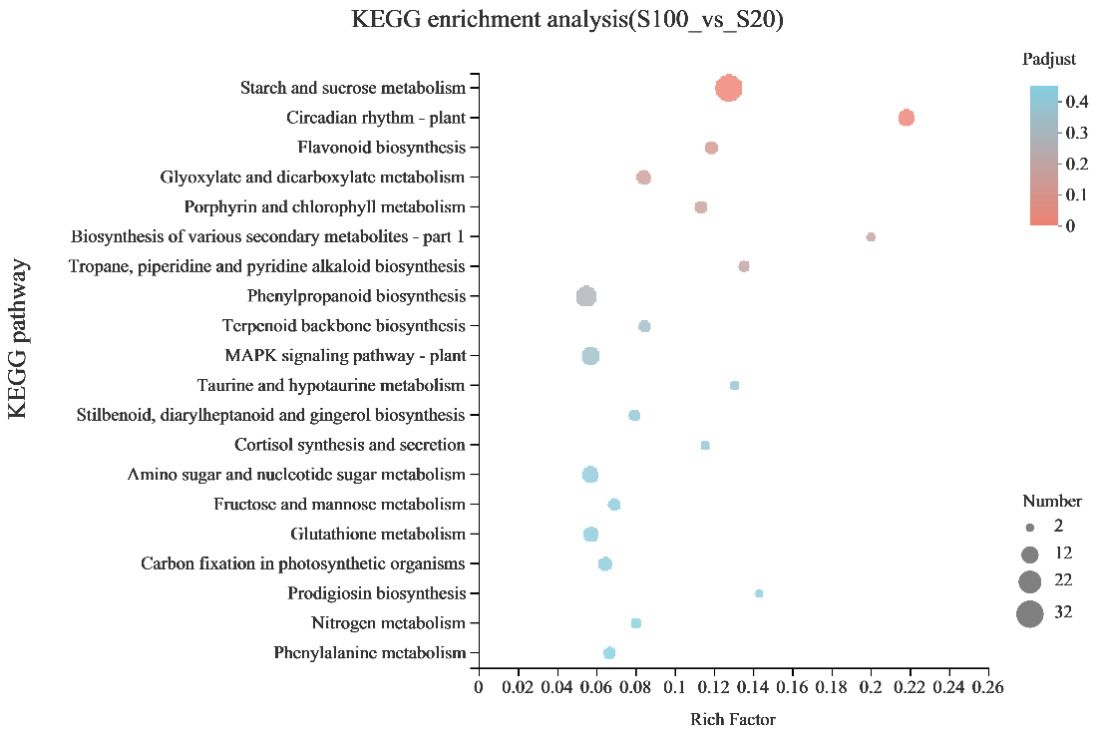
KEGG enrichment analysis. The vertical axis is the pathway entry, and the horizontal axis represents the ratio of the enriched gene number (sample number) to the annotated gene number (background number) in the entry. The greater the rich factor, the greater the degree of enrichment. The size and color of the dots indicate the number of genes in this pathway and the corresponding *P-adjust* range, respectively. The figure shows the enrichment results of the top 20 pathways of *P-adjust* from small to large.

Taking *P-adjust* < 0.05 as the threshold, the DEGs were analyzed for KEGG pathways with significant enrichment. The results showed that two pathways were enriched, namely starch and sucrose metabolism (map00500) and plant circadian rhythm (map04712) (Figure 8). Combined with the results of GO enrichment analysis, it is speculated that the related processes of carbohydrate metabolism and response to water may be involved in the sucrose-induced formation and development of *OL* rhizomes. Among them, 32 genes were enriched in the carbohydrate transport metabolism KEGG pathway (Table S9), and these genes were mainly sucrose and starch synthase genes, such as *Os06g0160700* (*OsSSI*), *Os01g0919400* (*OsSPS1*), and *Os10g0189100* (*OspPGM*), indicating that high-concentration sucrose can be converted into starch for storage. In addition, there were a few genes related to cell wall components, such as endoglucanase (*Os09g0530250*).

### 2.5. Quantitative Real-Time PCR (qPCR) Validation

Among the differentially expressed genes, 14 genes were selected for qPCR. The expression level of both the qPCR and the RNA-Seq results of the control group S20 were assigned as 1, and the relative expression level of the treatment group S100 was calculated. The results showed that the up- and down-regulation of gene expression detected by qPCR was consistent with the results of RNA-Seq (Figure S3).

**Figure 9:**
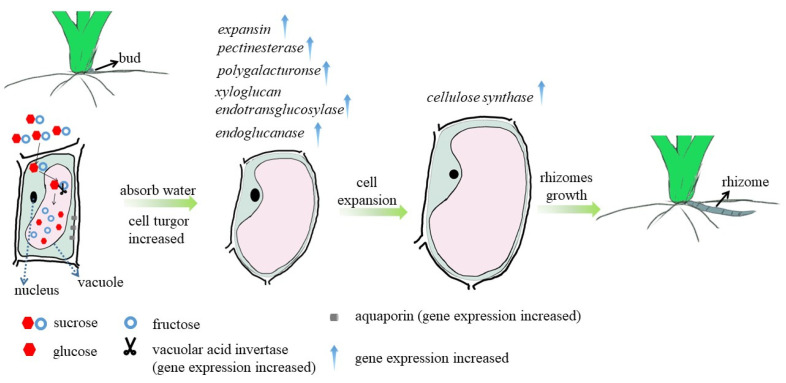
The regulatory mechanism of sucrose on rhizome elongation of *Oryza longistaminata*.

According to the above results, it is inferred that with the increase of sucrose concentration in the medium, the absorption of sucrose by *OL* increases. After sucrose enters the cell vacuole, it is decomposed into glucose and fructose by vacuolar acid invertase, which increases the cell osmotic pressure, and promotes the cell to absorb water and swell, causing the expression of genes involved in remodeling of plant cell walls, such as expansin, and then cell elongation. The final result is to promote the elongation of rhizomes in *OL* (Figure 9).

## 3. Discussion

Sucrose is the main product of photosynthesis and the main transport form of carbohydrates in plants, and it plays an important role in the entire life cycle of plants. Sugar is the main source of nutrients required for the growth of plant axillary buds [15], and elevated levels of sucrose promote the growth of buds [16]. An adequate supply of sucrose can reduce the competition of apical buds for sucrose, breaking the apical dominance, and facilitate the sprouting of lateral buds [17]. In addition, sucrose can also act as an osmotic agent, which can induce a certain degree of osmotic stress [18].

In this study, it was observed that when plants were transferred to a sucrose gradient medium, with the increase of culture time to about one month, phenotypic differences appeared in plants cultured at different concentration gradients. The 20 g/L and 40 g/L sucrose treatments had no rhizome formation, 60–100 g/L sucrose treatment had rhizome formation, and 120–160 g/L sucrose treatment had unhealthy plant growth. With the further extension of the culture time, even up to 3 months, the axillary buds of the plants in the 20 g/L and 40 g/L sucrose treatment groups still did not germinate or germinated individually, and no rhizomes were formed. The rhizomes in the 60–100 g/L sucrose treatment group grew and developed to the wall of the triangular flask, and began to grow up along the bottle wall, growing true leaves, and developing into ramets. In the 120–160 g/L sucrose treatment group, the leaves of the plants turned yellow, and some plants died. It shows that a certain concentration of sucrose is beneficial to the development of rhizome. However, too high sucrose concentration formed stress on the plant, resulting in a plant that could not grow healthily.

In *OL* rhizomes, the content of sucrose is similar to the concentration pattern of starch, decreasing gradually from the base to the tip of the rhizome [6], which indicates that the accumulation of sugar in the rhizome increases with the rhizome growth and development. By adding sucrose to the culture medium, the negative gravity of the *OL* rhizome is inhibited and the horizontal growth is promoted [6,11]. Horizontal growth of the stolon of *Cynodon dactylon* and *Paspalum vaginatum Swartz* is also promoted when sucrose supply is sufficient [19,20]. It was shown that sucrose could weaken the negative gravity response of both stolons and rhizomes, and high levels of sucrose are beneficial to the horizontal growth of rhizomes. It is suggested that sucrose plays an important role in the formation and development of rhizomes.

In this study, the expression of the vacuolar invertase gene *OsINV3* was up-regulated in the high-sucrose group (Tables S6 and S7), and the action of vacuolar acid invertase promoted the degradation of sucrose into glucose and fructose [21,22], resulting in an increase in the content of osmotic regulators and an increase in vacuolar osmotic potential, and also an up-regulation of cell aquaporin gene expression. High water channel activity of aquaporin [23], which promoted the water absorption and expansion of cells, thereby promotes cell elongation [24]. During the process of cell swelling and water absorption, the cell wall relaxed and remodeled, and the related genes involved in cell wall relaxation and remodeling were differently expressed (Table S6). Among them, the expression of expansin gene, polygalacturonase/pectin degrading enzyme genes and xyloglucan endoglucan glycosylase/hydrolase genes increased, and the expression of pectin methylesterase inhibitor gene decreased (Table S6).

Expansin, also known as a cell wall relaxation protein, is involved in various functions, such as plant stem elongation and water stress [25,26,27,28]. Expansins are able to weaken or break the non-covalent bonds between cell wall polysaccharides, causing hemicellulose to move, thereby causing cell wall loosening and, ultimately, cell wall stretching [29]. Both xyloglucan polygalacturonase/pectin degrading enzymes [30] are able to degrade pectin, which is the main polysaccharide in the primary cell wall of plants and is involved in cell wall plasticity [31,32]. The balance between pectin methylesterase (PME) activity and inhibition of post-transcriptional PME by pectin methylesterase inhibitors determines pectin methylesterification in plant cells [33]. *OsPMEI28* functions as a key component in regulating the level of methyl esterification of pectin, and the impaired methyl esterification level of pectin affects the biochemical properties of cell wall composition and leads to abnormal cell elongation in rice stem tissue and dwarf plants [33]. Xyloglucan endoglucan glycosylase/hydrolase catalyzes the hydrolysis or transfer of xyloglucan molecules and plays an important role in plant cell wall extensibility [34]. The action of these proteins leads to cell wall remodeling and elongation, cell elongation and, ultimately, rhizome elongation.

RNA-Seq also detected that the sucrose phosphate synthase gene *OsSPS1*, the starch synthase genes *OsSSI* were up-regulated in the high sucrose concentration group. Sucrose phosphate synthase is one of the key enzymes controlling sucrose synthesis in higher plants [35]. Starch synthases play a role in plant starch synthesis and accumulation [36,37]. Cytoplasmic fructose-1,6-bisphosphatase is an important enzyme in the sucrose synthesis pathway, and its lack of activity leads to insufficient sucrose supply, resulting in the inhibition of tiller growth in rice mutants of this gene [38]. It is speculated that the sucrose entering the *OL* was metabolized and used as a carbon source and energy for the sprouting of axillary buds and the growth and development of rhizomes (Figure 9).

## 4. Materials and Methods

### 4.1. Plant Material

The plant material used was the aseptic seedlings from medium-germinated seeds of *OL* (No. WYD-108). WYD-108 was introduced from Senegal, Africa, and preserved in the National Germplasm Resource Nursery (wild rice) of China.

### 4.2. Method

#### 4.2.1. Culture of *OL* Seedlings

The seeds of *OL* were collected by bagged selfing to avoid outcrossing, and were treated at 50 °C for 5 days to break dormancy. The dehulled seeds were disinfected with 0.15% mercuric chloride for 15–20 min, and then rinsed with sterilized distilled water 5 times [39]. The seeds were then transferred to the rooting medium until the 3–4 leaf stage at 26 °C, with 12 h light/12 h dark cycle [39]. The light intensity was about 140 μmol/m^2^/s. It took about 18 days for the seeds to grow to the 3–4 leaf stage on the rooting medium. The rooting medium was prepared based on 1/2 MS basic medium with 20 g/L sucrose added [40]. The basic medium formula is shown in Table S1.

#### 4.2.2. Culture of *OL* with Different Concentrations of Sucrose

Under aseptic conditions, the 3–4 leaf-stage *OL* seedlings were pulled out from the rooting medium and most leaves and roots were cut off, keeping the youngest ones. Then, the seedlings were transplanted to solid basic medium with different sucrose concentrations (sucrose concentrations of 20 g/L, 40 g/L, 60 g/L, 80 g/L, 100 g/L, 120 g/L, 140 g/L and 160 g/L), and cultured under 26 °C, 12 h light/12 h dark cycle, with light intensity 140 μmol/m^2^/s. Although rhizomes and tillers can develop from axillary buds, the morphology of rhizome buds is completely different from that of tiller buds. Rhizome bud in *OL* is onion-like-shaped and develops in an oblique direction [41]. In addition, the leaf morphology of tillers and rhizomes is also different. The tiller leaves are composed of a distal leaf and a proximal leaf sheath, while the formation of rhizome leaves is suppressed, and most rhizome leaves are wrapped by leaf sheaths [42]. These phenotypes can help us distinguish between tillers and rhizomes.

#### 4.2.3. Sample Collection for Transcriptome Analysis

Based on our initial results, the axillary buds of *OL* did not sprout under 20 g/L sucrose, and then 0.2 mg/L uniconazole was added into the medium to ensure sprouting of the axillary bud [12]. Although the axillary bud of *OL* can sprout spontaneously under 100 g/L sucrose, 0.2 mg/L uniconazole was also added to control the experimental parameters. The *OL* seedlings at the 3–4 leaf stage were transplanted into these two sucrose–uniconazole media with only the sucrose differing (100 g/L vs. 20 g/L). After the sprouting of axillary buds, the crowns (which are the shortened internodes at the base of the stem of grass species, also called shortened basal internodes), together with attached axillary buds, were quickly cut and placed into liquid nitrogen, numbered S20 and S100, respectively. Three biological replicates were sampled for both S20 and S100. All collected samples were stored at −80 °C. RNA isolation, library construction and sequencing were performed by Shanghai Majorbio Co., Ltd. (Shanghai, China). The sequenced data were uploaded in NCBI (the accession numbers is PRJNA894815).

### 4.3. Data Processing and Reads Alignment

After removing the adapter sequence, low-quality reads, sequences with high N rate (N represents uncertain base) and those too short raw reads, the quality control sequences (clean reads) were obtained. The clean reads were aligned with the reference genome (*Oryza sativa Japonica* Group (IRGSP-1.0), http://plants.ensembl.org/Oryza_sativa/Info/Index (accessed on 25 September 2022)). Mapped reads were assembled and spliced using the software StringTie (http://ccb.jhu.edu/software/stringtie/ (accessed on 25 September 2022)) and compared with known transcripts.

### 4.4. Abundance Estimation and Correlation Analysis

RSEM software (http://deweylab.github.io/RSEM/ (accessed on 25 September 2022)) [43] was used to quantify gene abundances. The expression level of each transcript was calculated according to the transcripts per million reads (TPM) method. Based on the normalized expression levels, the Pearson correlation coefficient between any two biological replicates was calculated.

### 4.5. GO and KEGG Ontology Enrichment Analysis

Using TPM as a quantitative indicator of gene/transcript expression, the genes with significant differential expression (DEGs) between groups were identified using DESeq2 software [44], with|log_2_FC| ≥ 1 and *p-adj* < 0.05 as the threshold. The DEGs were functionally annotated using COG (cluster of orthologous groups), GO (gene ontology) and KEGG (Kyoto Encyclopedia of Genes and Genomes). Taking *P-adj* < 0.05 as the threshold, the software Goatools (https://github.com/tanghaibao/GOatools (accessed on 25 September 2022)) was used to perform GO enrichment analysis. KEGG enrichment was performed using R-Package.

### 4.6. Quantitative Real-Time PCR Validation

The expression abundance of 14 DEGs randomly selected was evaluated by quantitative real-time PCR (qPCR). Total RNAs were reverse transcribed into cDNA using RT kit (PrimeScript^TM^ RT reagent Kit with gDNA Eraser, TaKaRa). qPCR analysis was performed on CFX96^TM^ Real-Time System (Bio-Rad, Hercules, CA, USA) with SYBR Mix (ChamQ Universal SYBR qPCR Master Mix, Vazyme, Nanjing, China). *Ubiquitin* gene (*LOC_Os03g13170*) was used as the reference gene, and the relative expression level of target genes was calculated according to the 2^−ΔΔCt^ method [45]. Primers are listed in Table S2.

## Data Availability

The datasets supporting the conclusions of this article are included within the article (and its Supplementary Materials).

## Supplementary Materials

The following supporting information can be downloaded at: https://www.mdpi.com/article/10.3390/ijms232113396/s1.
