# Supplementary material for: Sucrose Facilitates Rhizome Development of Perennial Rice (Oryza longistaminata)
Source: Int J Mol Sci. 2022 Nov 2;23(21):13396. doi: 10.3390/ijms232113396 (PMC9654561; doi:10.3390/ijms232113396)
Supplement: Supplementary file 1 [file ijms-23-13396-s001.zip › ijms-1966856_supp_to_conv.pdf]

International Journal of *Molecular Sciences*

MDPI

Article: Supplementary figures

Sucrose Facilitates Rhizome Development of Perennial Rice (*Oryza longistaminata*)

Zhiquan Fan †, Guanwen Huang †, Yourong Fan * and Jiangyi Yang *

State Key Laboratory for Conservation and Utilization of Subtropical Agro-Bioresources, College of Life Science and Technology, Guangxi University, Nanning 530004, China
* Correspondence: yrfan@gxu.edu.cn or fanyourred@163.com (Y.F.); yangjy@gxu.edu.cn or yangjy598@163.com (J.Y.)
† These authors contributed equally to this work.

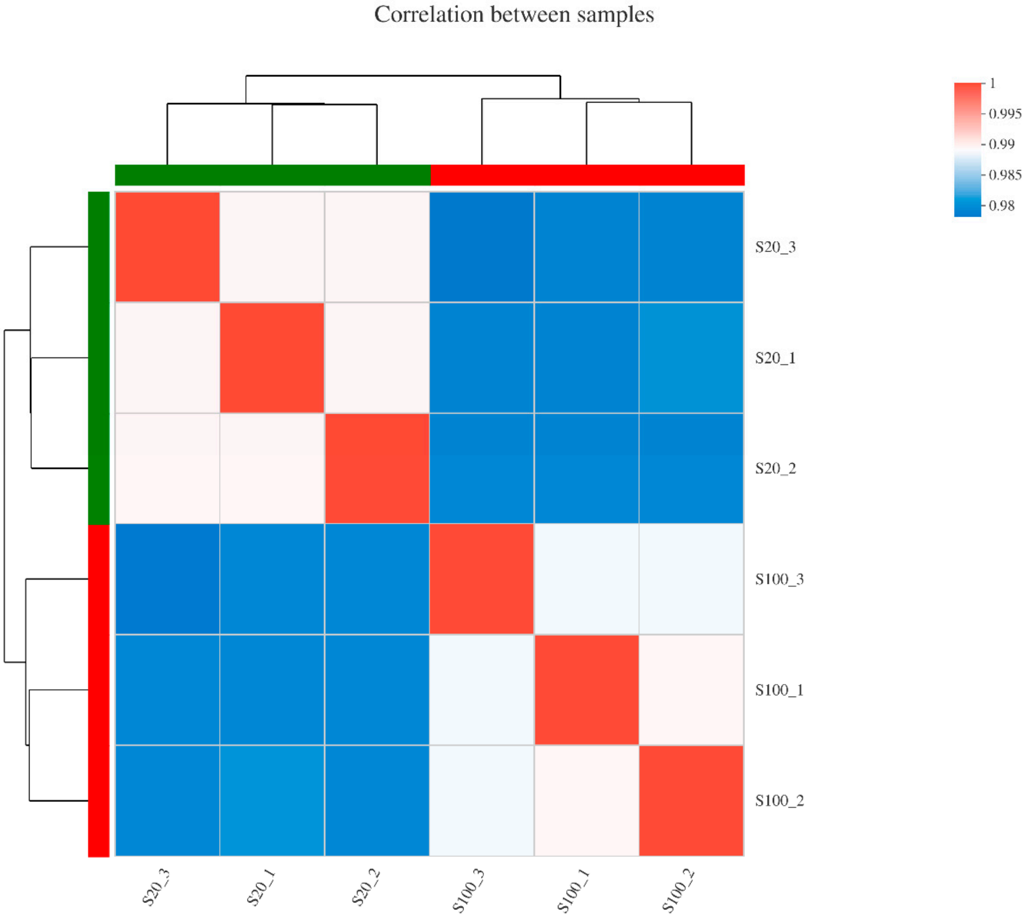

Figure S1. Cluster analysis of biological replication.

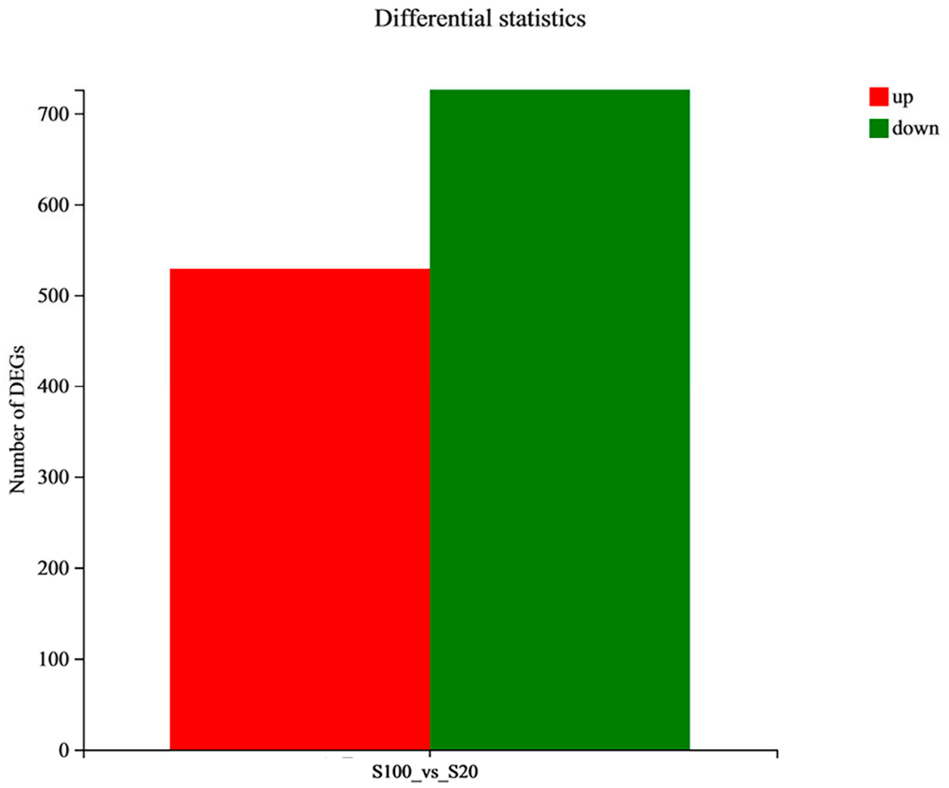

Figure S2. Statistics of differentially expressed genes.

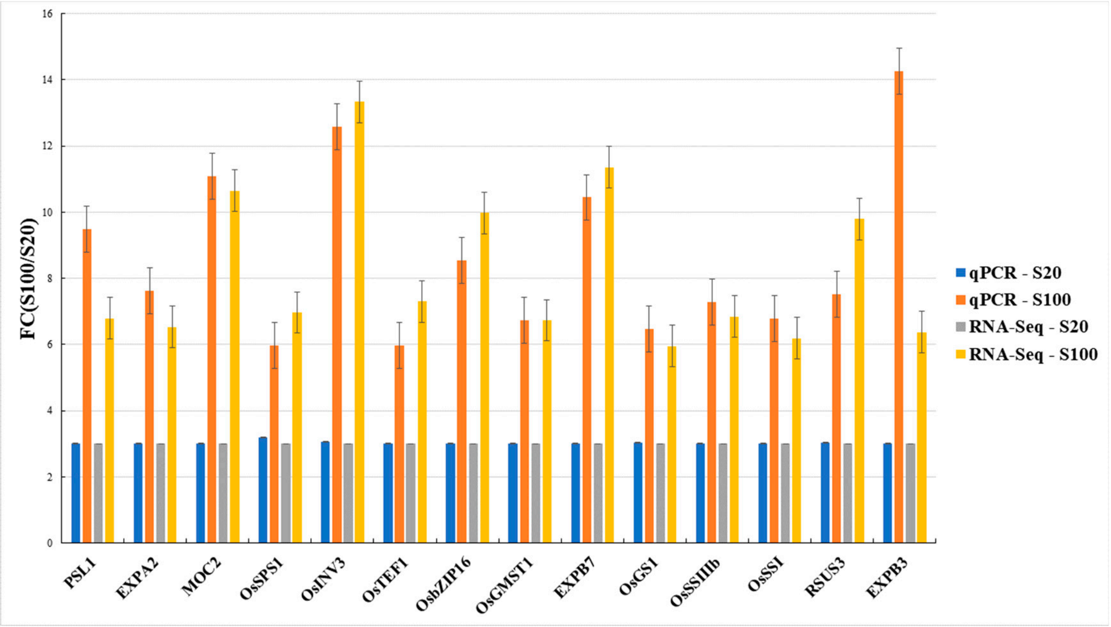

Figure S3. Quantitative real-time PCR validation.
